# Oligomer logic of memory molecules

**DOI:** 10.1186/s13041-026-01307-0

**Published:** 2026-05-08

**Authors:** Jerry W. Rudy

**Affiliations:** Department of Psychology and Neuroscience, University of Colorado, Boulder, CO USA

**Keywords:** PKMζ, PKM-zeta, KIBRA, WWC1

## Abstract

It has been 40 years since Francis Crick [1] noted the problem molecular turnover poses for maintaining memories and offered a general solution. The solution requires that the critical molecules must be replaced without altering the overall structure of the complex. It is timely then that Todd Sacktor’s group [2] has identified critical intermolecular interactions that satisfy Crick’s requirement. Sacktor’s early work identified the continuously active kinase, protein kinase Mzeta (PKMzeta) as a critical molecule for maintaining localized postsynaptic AMPA receptors that support long-term potentiation (LTP) and memory. More recent work revealed that PKMzeta forms heterodimers with the scaffolding protein KIBRA (KIbra BRAin) and preventing dimerization erased both LTP and memory. Even so, dimers degrade too fast to support long-lasting memories. Based on biophysical modeling, Sacktor’s group with Harel Shouval reasoned that if KIBRA-PKMzeta heterodimers interact to form oligomers (such as hexamers), they can survive molecular turnover because as a dimer degrades it can be replaced by another. AlphaFold 3 predicted a site where the small molecule inhibitor, zeta-stat, would bind and disrupt oligomer formation. If so, then infusing zeta-stat into the hippocampus should erase long-term memory. This predicted outcome was observed. Thus, Crick’s solution has been achieved. Oligomers formed from KIBRA-PKMzeta dimers allow degraded individual molecules to be replaced one at a time while maintaining their overall structure. This permits a continuous presence of PKMzeta where it interacts with AMPA receptors (through GluA2 subunits) and other molecules to ensure long-term memories endure.

## Introduction

When Francis Crick [1] called attention to the problem molecular turnover presents for memory, he suggested that it could be solved if “…molecules in the synapse interact in such a way that they can be replaced with new material, one at a time, without altering the overall state of the structure”. When he proposed this general solution almost nothing was known about the molecular underpinnings of memory. However, with the discovery of long-term potentiation (LTP) and technical advances that permit the manipulation and imaging of synaptic molecules, significant progress has been made toward understanding how memories are created and survive molecular turnover. In this issue Todd Sacktor and his colleagues [2] provide an important advance in our understanding of how the molecular turnover problem is solved. To appreciate this contribution, it is helpful to discuss three important questions raised by Crick’s in principle solution.

- What are the molecules that need to be replaced?
- What molecular interactions orchestrate replacement?
- What molecular interactions maintain the orchestrator?

## What are the molecules that need to be replaced?

**Fig. 1:**
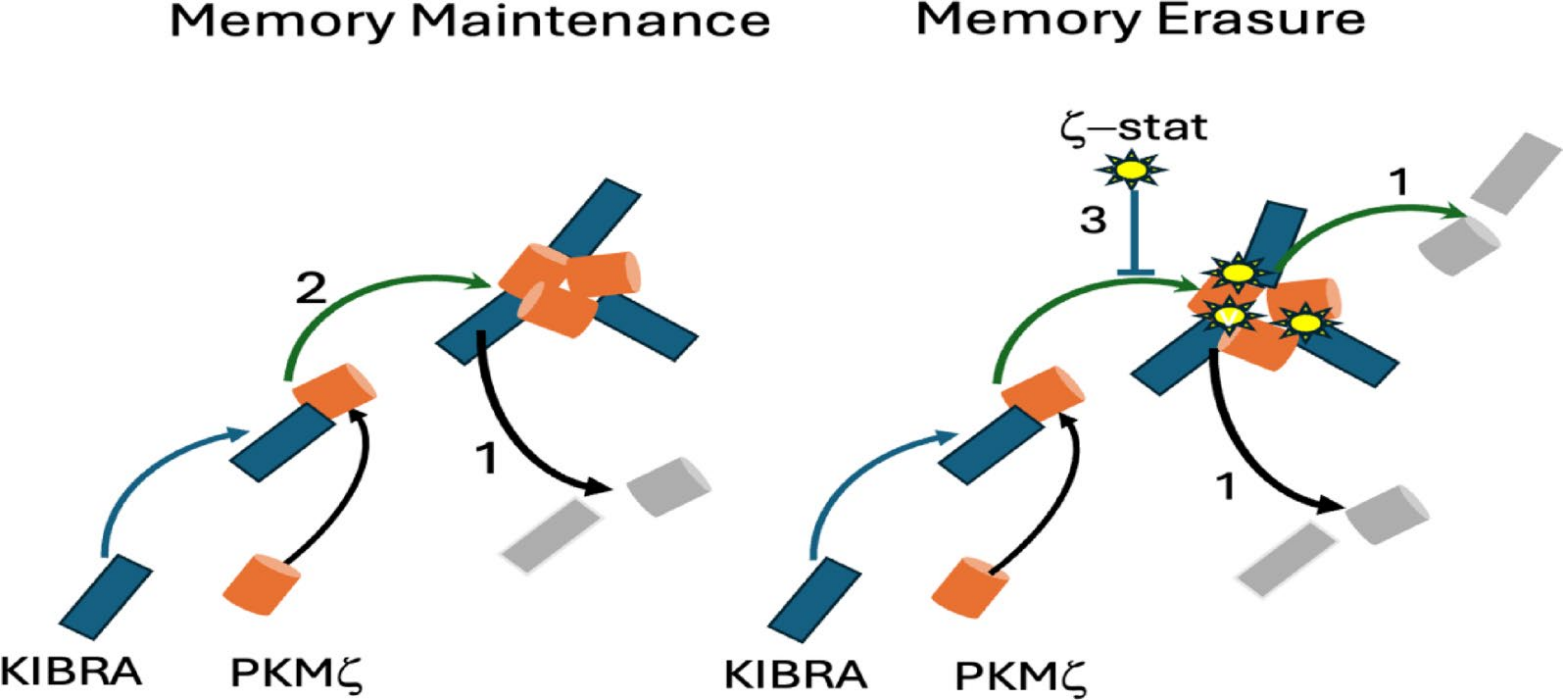
Left, by forming hexamers, memories are maintained because degraded KIBRA-PKMzeta (PKMz) heterodimers [1] can be replaced [2]. Right, Memories are erased because zeta-stat (z-stat) prevents the replacement of degraded heterodimers [3]

Before any flesh can be put on Crick’s proposal one must know the critical molecules that need to be replaced. This requires understanding the molecular endpoints produced by LTP induction that support memory. The abbreviated story is that glutamate released onto dendritic spines activates both NMDA and AMPA receptors to initiate calcium-dependent processes that generate a rapid reconstruction of the actin cytoskeleton and an *increase in the population of AMPA receptors* in the postsynaptic density [see Nicoll [3] for a detailed history]. The enhanced field potential that is the signature of LTP reflects increased sodium leaving the extracellular space to enter the cell through the AMPA receptors. Thus, minimally, it is the increase in the population of AMPA receptors that must be sustained to maintain memory (Fig. 1).

### What molecular interactions orchestrate replacement?

At around the time Crick pointed to the molecular turnover problem, John Lisman [4] independently raised the same issue and suggested that synaptic stability could result from “a kinase that is activated by phosphorylation and capable of intermolecular autophosphorylation.” Embedded in this idea is there is a molecular complex that can self-sustain indefinitely. Fortuitously, Mary Kennedy’s laboratory [5] discovered that the autophosphorylation of type II calcium/calmodulin-dependent kinase (CaMKII) rendered the enzyme’s activity independent of calcium/calmodulin. This was subsequently attributed to the ring-like holoenzyme structure of CaMKII, which allows its subunits to autophosphorylate and self-sustain. Given its many roles in establishing LTP, Lisman proposed CaMKII as a critical memory maintenance molecule. However, this appealing proposal did not hold up [6–8].

Yet, in the early 1990s protein kinase Mzeta (PKMzeta), a PKC isoform, with potential memory maintenance properties was discovered [see Sacktor [9] for historical perspective]. PKMzeta lacks a regulatory domain and is continuously active. It is rapidly translated in the dendritic spine regions when LTP is induced and once translated, PKMzeta can self-perpetuate by promoting its own local translation [10]. Critically, when perfused into CA1 pyramidal cells in CA1 slices it potentiates synapses, and when it is inhibited many hours after induction, LTP is erased [11–13]. Moreover, it does this by reconfiguring GluA2-dependent AMPA receptor trafficking [14–15].Numerous papers report that it is critical to maintaining LTP and memory [11–18].

Armin Schneider’s group [19], however, reported that PKMzeta stability depends on the scaffolding protein KIBRA (KIdney BRain). In the absence of KIBRA, PKMzeta levels rapidly decreased, and this is accompanied by a memory impairment. Thus, PKMzeta’s self-replicating properties alone cannot maintain long-lasting memories.

## What molecular interactions maintain the orchestrator?

This takes us to the significance of the Sacktor group’s report [2]. PKMzeta alone degrades too quickly to support long-term memory. That KIBRA-PKMzeta interactions must be part of the solution was recently confirmed by Sacktor’s group [20], who reported that KIBRA and PKMzeta form heterodimers when LTP is induced, and if their interaction is prevented or disrupted both enduring LTP and long-term memories are lost. However, even dimerization alone cannot support memory beyond a few days because the dimers will degrade. Thus, other intermolecular interactions must be involved. Biophysical modeling [21] indicated that if KIBRA-PKMzeta heterodimers interact to form larger oligomers (such as hexamers) they can survive molecular turnover. This is because degraded individual molecules of the hexamer can be replaced within the complex by newly synthesized KIBRA and PKMzeta.

To test this hypothesis Sacktor’s group relied on AlphaFold3 to locate potential sites on these proteins that are critical to the formation of the KIBRA-PKMzeta oligomers. The small molecule zeta*-*stat was previously used to successfully inhibit KIBRA-PKMzeta interactions [20]. Importantly, the zeta*-*stat binding site on PKMzeta was not identified as a site for heterodimer formation. Thus, perfusion of the drug would not prevent dimerization. However, it was identified as a site where a KIBRA-PKMzeta heterodimer interacts with both a second KIBRA and another PKMzeta, which should be critical for oligomer formation. They predicted that perfusing zeta*-*stat into the hippocampus would prevent oligomer formation, and therefore should erase a 30-day old memory. This result was obtained.

Thus, Crick’s and Lisman’s in principle solution has been achieved. Oligomers formed from KIBRA-PKMzeta dimers allow degraded individual molecules to be replaced one at a time while maintaining their overall structure. This allows a continuous presence of PKMzeta where it interacts with AMPA receptors (through GluA2s) and other molecules to ensure memories endure.

Beyond its scientific accomplishment, this discovery offers potential insights into memory disorders. For example, it has recently been reported that memory impairment associated with pathogenic tau could be restored by introducing a piece of KIBRA into the brain, and this stabilized the presence of PKMzeta [22]. Stay tuned!

## Data Availability

No datasets were generated or analysed during the current study.
